# How do people conceptualise the reuse of medicines? An interview study

**DOI:** 10.1111/ijpp.12391

**Published:** 2017-08-09

**Authors:** Hamza Alhamad, Nilesh Patel, Parastou Donyai

**Affiliations:** ^1^ Department of Pharmacy University of Reading Reading Berkshire UK

**Keywords:** medicine reuse, medicine waste, thematic analysis, theory of planned behaviour

## Abstract

**Objectives:**

To capture people's beliefs about medicines reuse and to map the determinants of intentions to reuse medicines in the future.

**Methods:**

Participants were recruited through an advert placed in the university's community newsletter reaching 15 000 households. Adults wishing to participate were interviewed using convenience sampling, with recruitment continuing until data saturation. Participants were interviewed face‐to‐face by two researchers using a semi‐structured interview schedule based on the theory of planned behaviour (TPB). Interview transcripts were analysed by thematic analysis, with the themes classified according to the TPB. The University's research ethics committee approval was obtained.

**Key findings:**

Nineteen participants were interviewed. The potential economic and environmental benefits of medicines reuse were juxtaposed with stability and safety worries. Participants trusted pharmacists to quality‐assure returned medicines, but wondered if they had the time and storage space to dedicate to medicines reuse. Environmentalists were seen as the main proponents of medicines reuse with drug manufacturers, some taxpayers and parents seen as the main opponents. The physical characteristics of reused medicines, and quality assurance and logistics of reuse processes were seen to enable/obstruct engagement in medicines reuse. A working definition of medicines reuse as a behaviour was developed.

**Conclusions:**

People could potentially agree to reuse medicines if their concerns are addressed and the process is well defined and managed. This is a qualitative study with a small number of participants meaning the results may not be generalisable. The themes generated will enable a structured questionnaire to be developed for quantifying broader views.

## Introduction

This article relates to the idea that unused prescribed medication returned by one patient to a pharmacy can be dispensed and therefore reused by another patient (‘medication reuse’) as a strategy for reducing medicinal waste in the United Kingdom (UK). NHS England defines medicinal waste as ‘Any substance or object the holder discards, intends to discard or is required to discard’^1^ and the World Health Organisation further specifies medicinal waste as ‘expired, unused, spilt and contaminated pharmaceutical products, drugs, vaccines and sera’.^2^ In the UK if a prescribed medication is no longer being used, then conceptually that medication is medicinal waste because it ought to be discarded rather than, say, used by another patient. Medicines that have been dispensed to patients, even if unused, are not currently allowed to re‐enter the pharmaceutical supply chain. One technical reason is uncertainty about the biochemical integrity of medicines on leaving the formal distribution chain; for example, storage conditions in a patient's home may degrade the active ingredients. The potential for counterfeit medicines to enter the pharmaceutical supply chain is another concern.

The financial cost of medicinal waste in the UK is estimated as £300 million per year for prescribed medication.^3^ However, monetary cost is only part of the burden of medicinal waste. Environmental costs are also a concern as the presence of pharmaceuticals in the environment increases, with inappropriate disposal of medicinal waste potentially contributing. Research has found that people are more likely to dispose of a range of unwanted prescribed medicines in common refuse or down the sink/toilet than return these to pharmacies for correct disposal.^4^, ^5^ The environmental burden is not inconsequential in that other studies have documented the possible emergence of antibiotic resistance in wastewater.^6^ Prescribed medicinal waste can also impact negatively on the environment through the ‘carbon footprint’. Therefore logically, reducing medicinal waste relating to unused prescribed medicines could impact on environmental as well as financial costs.^7^, ^8^

NHS England categorises prescribed medication waste as non‐adherence behaviours, preventable causes (e.g. patient stockpiles) and non‐preventable causes (e.g. patient dies, recovers or treatment is changed).^1^ To reduce medicinal waste, one approach is to prevent waste in the first place. Preventing waste is at the top of the Waste Hierarchy, a grading system which ‘ranks waste management options according to what is best for the environment’, with ‘prepare for reuse’, ‘recycle’, ‘other recovery’ and ‘disposal’ following ‘prevention’ in decreasing order of preference.^9^ Interventions that try to *prevent* medicinal waste are not always effective and paradoxically, the most common causes of medicinal waste are non‐preventable.^10^ *Reuse* and *recycle* remain largely unexplored because unused medicines are not currently permitted to be reused in the UK. An inhaler recycling scheme has been trialled in Brighton, but this focussed on collecting and *recycling* the inhaler device rather than recovering the medicinal product contained in the inhaler canister.^11^ Medicines returned to a pharmacy are automatically considered to be waste that requires appropriate *disposal*. Therefore, what normally takes place in community pharmacy practice sits at the foot of the Waste Hierarchy. Yet, anecdotally patients returning their medicines to pharmacies often voice a wish for these to be reused by others. In fact, an NHS sustainability survey carried out by Ipsos MORI in 2011 reported half of the respondents as likely to accept re‐issued medicines returned to pharmacies.^12^

A formal, quality‐assured system for collecting and reusing unused prescribed medicines could provide an effective solution for the problem of medicinal waste in the UK because it has the potential to address both the preventable and non‐preventable causes of medicinal waste, which other management options cannot address. There is precedence of medication reuse in other countries. For example, in the United States unused medicines are collected and redistributed to patients who are less able to afford the cost of medication.^13^ Because the implementation of medicines reuse in the UK would rely heavily on people's uptake of this idea, we have set out to develop an understanding of what the public thinks about this concept. To date, no formal research study has examined the general public's views about and openness to the idea of medicines reuse, although one study does exist that focuses on pharmacists’ views.^14^ The aim of the current research was to capture people's beliefs about medicines reuse and to map the determinants of people's intentions to take part in medicines reuse behaviour. The research question was ‘what are the behavioural determinants of medicines reuse?’ The objectives were to define medicines reuse as a behaviour and identify beliefs about this behaviour using qualitative interviews and the theory of planned behaviour (TPB).^15^, ^16^

## Methods

### Compliance with ethical standards

This study was approved by the University of Reading's Research Ethics Committee through the School Exemptions process (reference number 30/15) on 6/5/2015. Written consent from each participant was obtained before the interviews.

#### Approach

The aim was to capture people's beliefs about the idea of reusing medicines and to identify the relevant behavioural determinants within a health psychology paradigm. Thematic analysis was carried out because it provides a way of organising qualitative interview data in the form of themes: recurrent topics, ideas or statements identified across the corpus of data. Thematic analysis also allows for these themes to be mapped against a theoretical framework within a deductive approach.^17^ The framework of the TPB was used to identify the themes.

**Figure 1:**
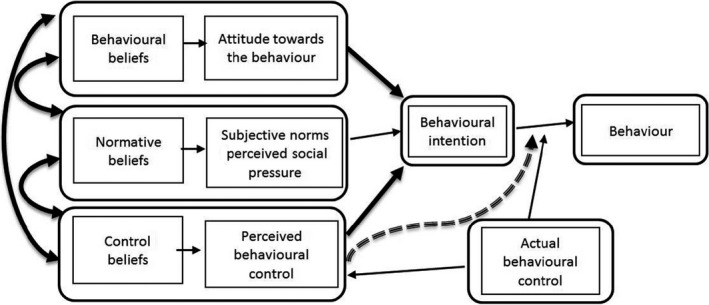
Schematic representation of the theory of planned behaviour, adapted from Ajzen (2006)^15^, showing the relationship between the determinants of behaviour (copyright ©2006 Icek Ajzen).

The TPB makes a distinction between behaviour and behavioural intentions on the basis that what people intend to do is more predictable than what they will actually do.^15^ Accordingly, behavioural intentions are a function of three determinants: firstly, the person's attitude in terms of likely consequences of the behaviour (behavioural beliefs), that is the individual's positive or negative evaluation of taking part in the behaviour, creating a favourable or unfavourable *attitude towards the behaviour;* secondly, the person's beliefs about the normative expectations of other people (normative beliefs), that is social pressure to take part or not take part in the particular behaviour, creating a *perceived social pressure* or subjective norm; thirdly, the individual's beliefs about the existence of factors that may enable or obstruct taking part in the behaviour (control beliefs), that is whether the person has control over the behaviour, creating a belief about *perceived behavioural control*. The combination of these three factors leads to the formation of an individual's *behavioural intention*, which is thought to be the immediate antecedent of the behaviour according to the TPB. With a sufficient degree of actual control over the behaviour, the model expects that people would carry out their intentions when the opportunity arises (Figure 1).

#### Setting and participant recruitment

Participants were recruited in spring 2016 through an advertisement placed in the university's community newsletter circulated biannually to local residents. The university's community newsletter is often used to recruit participants to research projects because it reaches 15 000 local households. The advert used for this study sought English‐speaking adults with an interest in the concept of medicine reuse and willingness to participate in a qualitative study by attending an interview at the university campus. Medicines reuse was defined as ‘the idea that medication returned by one patient can be dispensed by a pharmacist to another patient (instead of disposal as waste – which is what currently takes place)’ – see Appendix S1 for a copy of the advert. Participants either contacted the research team directly or were introduced to the research team via already‐recruited participants via email. A balanced number of men and women were interviewed, and there was also good representation across different age bands meaning that recruitment continued until data saturation using convenience sampling. Data saturation was guided by an initial desired sample size (*n* = 20) determined by PD and HA according to the TPB methodology^15^ which was modified down when no additional themes were identified after interviewing the 15th participant.^18^ After this time point, four more people were interviewed but three additional people who contacted the research team expressing an interest were turned away.

#### Data collection

A semi‐structured interview schedule based on the TPB and focussing on behavioural beliefs, normative beliefs and control beliefs in relation to medicines reuse was constructed and used in the interviews (see Table S1).^15^ Fifteen participants were interviewed by the main author who is an experienced researcher (PD) with another author (HA) (a PhD student) in attendance, after which the remaining four participants were interviewed by HA. Written consent was obtained, and the interviews, which lasted around 40 min, were audio‐recorded. Participants were recruited until no more new and significant concepts emerged (i.e. sampling saturation).

#### Data analysis

Interviews were transcribed verbatim, password‐protected and anonymised/de‐identified by ‘*The Transcription Agency*’, a university‐approved supplier. HA reviewed all transcripts to confirm that names or other information that might identify the participants had been removed, and he also ensured data integrity by cross‐checking the transcripts against the interview recordings, in consultation with PD. The interview transcripts were analysed manually, and then the NVivo 10 software (QSR International Pty Ltd. Version 10, 2012) was used to visualise theme connections and to construct the final thematic map. The thematic analysis process was carried out by HA according to the six phases described by Braun and Clarke^17^ and was reviewed by PD. The recordings were read and re‐read before being coded and categorised according to the TPB to define behavioural beliefs, normative beliefs and control beliefs about the reuse of unused prescribed medicines.

## Results

From 22 participants who contacted the research team, a total of 19 were recruited (11 female), including one couple who were interviewed jointly. Two participants were British Asian, and 17 were White British. Participant age groups were 40–49 (*n* = 3), 50–59 (*n* = 2), 60–69 (*n* = 8) and >70 (*n* = 6).

Three major categories were identified and labelled: ‘Consequences of medicines reuse’ (relating to behavioural beliefs), ‘Exemplar and anti‐exemplar individuals and groups’ (relating to normative beliefs) and ‘Expectations about returned medicines’ (relating to control beliefs). The compositional structure of these categories is described in Tables 1, 2, 3.

**Table 1:** The compositional structure of category 1 ‘Consequences of medicines reuse’

**Consequences of medicines reuse**
Participants’ attitudes towards medicines reuse involved an evaluation of the benefits and the risks associated with the distribution of returned medicines to other patients:
***Potential advantages of medicines reuse***
A. Economic impact on the NHS
Direct monetary savings for the NHS Reduction in manufacturing expenditure Cost‐benefit of reusing cheaper medicines
B. Environmental effects
Reduction in negative environmental effects of medicines disposed inappropriately Reduction in the carbon footprint
***Potential disadvantages of medicines reuse***
A. Poor quality medication
Temperature of storage Humidity of storage environment Cleanliness of the storage environment
B. Harmful medication
Deliberate or malicious tampering with returned medicines Medicines as a source of infection if contaminated
C. Incorrect medication
Errors introduced by patients Errors introduced by pharmacists Risk posed by accepting counterfeit medicines

**Table 2:** The compositional structure of category 2: ‘Exemplar and anti‐exemplar individuals and groups’

**Exemplar and anti‐exemplar individuals and groups**
The groups of individuals or people whom the participants thought would or would not engage with and approve of medicines reuse
***Individuals or groups of people who might approve of medicine reuse***
A. The Green movement
Spouses and partners, relatives and friends who ‘think green’ Environmentalists The Green Party, the political organisation
B. The elderly
Those with a dislike of waste and an affinity for frugality
***Individuals or groups of people who might disapprove of medicine reuse***
A. Pharmaceutical companies
Employees Beneficiaries
B. Taxpayers
UK Taxpayers with a sense of entitlement
C. Vulnerable patients (those making a decision for them)
Babies Children
D. The elderly
Cautious individuals worried about safety Terminally ill patients

**Table 3:** The compositional structure of category 3: ‘Expectations about returned medicines’

**Expectations about returned medicines**
Factors that may facilitate or impede the workability of medicines reuse for individuals
***Physical characteristics of returned medicines***
A. Original packaging of the medicine
Medicines sealed by the manufacturer potentially suitable to be reused Medicines in blister packaging potentially suitable to be reused
B. Whether the packaging had been opened or not
Only unopened and sealed medicines to be reused Medicines not sealed or with a broken seal not to be reused
C. Remaining shelf life of medication
Medicines should have more than 6 months of shelf life if to be reused
D. Pharmaceutical presentation (formulation) of the product
Solid oral dosage forms potentially suitable to be reused Liquid, creams and gels, and injections not to be reused
***The quality assurance of returned medicines***
A. Storage conditions
Temperature and humidity of storage environment and risk of degraded product Cleanliness of the storage environment and risk of spread of infection
B. Tampered product
Malicious damage to the product to be ruled out Accidental damage to the product to be ruled out
C. Counterfeit medicines
Medicines bought from untrusted sources including online sources not to be reused
***The logistics of medicine reuse***
A. Collection and redistribution of returned medicine ‘on‐site’ within a pharmacy setting
Efficiency of system for returning medicines Space for collection, processing and storage of returned medicines Pharmacists’ time availability to conduct quality assurance of returned medicines
B. Collection and redistribution of returned medicines ‘off‐site’
Collection spots within pharmacies Clinical centres responsible for processing medicines for reuse Pharmaceutical companies to be involved in funding and supporting reuse processes
C. Incentives for taking part in medicines reuse
Points reward system to encourage the return of medicines Discount on medicines to encourage the reuse of medicines

Participants interviewed in this study were generally in favour of the idea of medicines reuse in that they felt the NHS should move to a system whereby unused prescribed medicines would be reused instead of being discarded. This system of reusing prescribed medicines would not be obligatory, with patients opting in or out, and the whole process regulated to prevent misuse. The following quote illustrates this point:

Medicine reuse should be regulated and monitored by NHS to avoid the risk of having black market, this include pharmacist selling the collected medicines online, and also counterfeit medicines that patient bought online should not put back the shelf (if returned) and this will be assured during a quality check by the pharmacist. (P17, female, >70 age group)

### Consequences of medicines reuse

This category encapsulates participants’ understanding of the advantages and disadvantages of medicines reuse if ever implemented (see Table 1).

#### Potential advantages of medicines reuse

Both economic and environmental advantages of reusing medicines were discussed. Some perceived that reusing unused medicines would save money for the NHS and reduce manufacturing costs by cutting medicinal waste. The following quote exemplifies this point:

I would say the main advantage of reusing medicines is saving on cost, in this country masses of drugs are wasted. When you have been prescribed something and did not need much of it, and then you think what an awful waste? (P5, Male, 60–69 age group)

In addition, medicines reuse was thought more applicable for expensive medicines especially if logistical costs of reuse processes were to be substantially higher than the monetary value of cheaper medicines; logistical costs were conceptualised in different ways. For example, if medicines reuse processes could not happen in a pharmacy because of competing priorities or lack of storage space, a formal, costly system for collecting and despatching unused medicines to, say, a clinical centre might be needed; there technicians could work to check, repackage and prepare the medicines for reuse, which would carry a cost. The following quote illustrates the former point:

Generic medicines, maybe they are so cheap that a packet of aspirin cost maybe 16p or something, but maybe some of the more expensive medicines that is definitely worth reusing. (P3, male, 40–49 age group)

Medicines reuse was thought to reduce the proportion of medicines thrown into household bins and encourage people to return unused medicines to a pharmacy, thus helping reduce negative environmental effects arising from medicines reaching landfill or the water supply. Some felt knowing returned medicines were destined for disposal under the current system acted as a disincentive for returning unused medicines to a pharmacy. For example:

I think one of the reasons people put medicines down the loo is because they know if they take the medicine back to the pharmacist he is going to destroy them anyway so they think, why I should make the effort with this, pointless. They don't understand the damage they might be doing so I think there would be an environmental benefit. (P15, male, 50–59 age group)

Medicine reuse was thought to reduce the overall carbon footprint of medicines by impacting on manufacturing and transport of new medicines. For example:

So what I'm describing I think are people who are more aware, shall I say, of a bigger picture, they're not thinking just personally, they're thinking what can I do, does it save the environment, if one less packet of pills has to be made that's one less energy, that's less transport, it's all the good reasons, not just money. (P2, male, >70)

### Potential disadvantages of medicines reuse

Participants identified a range of issues with reusing medicines that had been in the hands of other people. The proper storage of unused medicines in terms of the temperature, humidity or cleanliness of the storage environment was one concern. Linked to this was the impact on the safety of unused medicines. Safety was conceptualised as inadvertent contamination or deliberate tampering. For example:

I think the main issue of reusing medicines would be the risk. I suppose some medications have to be stored at certain temperatures, like insulin. Also you would have to be assured that the medicine had not been tampered with. (P4, female, 60–69 age group)

In addition, the risk of medication errors was highlighted in terms of errors introduced by patients and the risk of returning counterfeit medicines. The risk of errors made by pharmacists was also a concern such as redistributing the wrong medicine to a patient and accepting counterfeit medicines. For example:

There could be a risk of medication error being made, for example if somebody put a medication back in the wrong box and returned it. There have to be very strict rules on checking the returned medicines. (P6, male, >70 age group)

Participants’ recognition of the advantages of medicines reuse was juxtaposed with assertions about a need for quality and safety assurances. Pharmacists were trusted to carry out quality and safety checks, but participants worried whether pharmacists had the time to devote to such assurances (detailed further in the section entitled ‘Expectations about medicines reuse’).

### Exemplar and anti‐exemplar individuals and groups

This category encapsulates participants’ understanding of individuals or groups of people who would partake or particularly engage with and promote medicines reuse (exemplar individuals and groups) and those who would not (anti‐exemplar individuals and groups) if a scheme were to be implemented in the future.

#### Individuals or group of people who might approve of medicine reuse

Those subscribing to the ideology of the ‘Green movement’ were considered to support medicines reuse, with spouses and partners, relatives and friends who *think green*, environmentalists and members of the Green Party, identified as people who might encourage medicines reuse. For example:

I think my husband and some friends, I think people who thinks green would support it. I would have thought most environmentalists would support it because the other things is, a lot of this stuff does end up in the water somehow or other, and affects wildlife. (P17, female, >70 age group)

#### Individuals or groups of people who might disapprove of medicine reuse

Pharmaceutical companies and their employees (or others with an interest in these companies) were considered amongst the group that would disapprove of medicines reuse because of a potential to reduce financial profits. For example:

I wonder if people working in pharmaceuticals would not frown upon it in some way if their profits are being affected. (P11, female, 40–49 age group)

Long‐standing taxpayers were another group who might disapprove of medicines reuse because of a sense of entitlement to receive ‘the genuine medicine’. For example:

Getting access to the NHS services is at the cost of the UK taxpayer. I think because it's so ingrained in this country, the NHS and the prescription process, that people almost feel that it is now like an entitlement to have the genuine medicine at a fixed cost, and that kind of thing. (P1, female, 60–69 age group)

Participants on the whole believed that people, especially mothers, may not approve of medicines reuse for their children, with babies particularly seen as a ‘very special group’. For example:

I think mothers are probably very cautious for their offspring, and wants the best for her child, there's a kind of feeling because it's brand new, off the shelf, it's purer, it's safer, there's no element of risk’. (P2, male, >70 age group)

Participants had contradicting thoughts regarding the stance taken by ‘the elderly’. Some thought older people would support reusing medicines because of a natural aversion to waste stemming from experiencing shortages around the Second World War; this was compared to a younger generation who might dislike using ‘second‐hand medicines’. For example:

I think particularly amongst the older generation would probably be more susceptible to saying, yeah medicine reuse is good idea, because we were brought up not to waste things. I do not know if youngsters think about that kind of thing as much because there is a surplus of everything these days but there was not when we grew up so we don't, we still don't waste things, we still mend things. (P17, female, >70 age group)

I think older people, the make do and mend generation who experienced shortages after Second World War, who are fast becoming rare and rarer. (P14, male, 60–69 age group)

Others thought that the elderly might in fact disapprove of medicines reuse if they have a terminal illness or might be more cautious and concerned about the safety of returned medicines.

### Expectations about returned medicines

This category encapsulates participants’ understanding of factors that may facilitate or impede the workability of medicines reuse as a formal process and is expressed in terms of the participants’ expectations about returned medicines (see Table 3).

#### Physical characteristics of returned medicines

It was clear that not all returned medicines were considered as suitable for medicines reuse. There was general agreement that reused medicines should be those originally packaged in sealed or in blister‐pack containers, be unopened, comprise of oral solid dosage forms only, be a genuine medicine (not a counterfeit) and have more than 6 months of shelf‐life remaining. In contrast, returned medicines that have a broken seal, have been opened, liquids and injectable medicines, controlled drugs, medicines with <6 months of shelf‐life remaining and medicines obtained from mistrusted or online sources would be excluded from the reuse process. For example:

I don't think medicine in a liquid form can be reused, someone might introduce something such as foreign body. This apply to gel and cream which is maybe easier to inject or get something in it, whereas in a blister pack you can tell whether it is been tampered with or not. (P7, female, 60–69 age group)

#### The quality assurance of returned medicines

In addition to physically checking returned medicines, there should be stringent quality and safety checks by the pharmacist, to confirm suitability for reuse. The checking process would involve the pharmacist confirming storage conditions and discounting any risk of product degradation, contamination or infection. The pharmacist would check that the product had not been tampered with, maliciously or accidentally, damaged, bought from an online source, and was not a counterfeit. For example:

I would be quite happy to reuse medicines as long as I know that the safeguards have been put in place that the returned medicines has not been tampered with. (P4, female, 60–69 age group)

#### The logistics of medicine reuse

The medicines reuse processes including the collection and the redistribution of returned medicines were considered in depth by the participants. Medicines could potentially be returned to pharmacies (community pharmacies, pharmacies within the GP clinics and hospital pharmacies) and assessed ‘on‐site’. Pharmacists were considered to be the professional group qualified to quality assure the suitability of returned medicines for reuse purposes. Potential challenges to an on‐site system were the pharmacist's availability for collecting and checking returned medicines, space within a pharmacy to enable processing and storage of returned medicines, and whether the process of returning medicines would be slick and rapid for patients (which was preferred to having to queue). For example:

As all returned medicine have to be checked. So this could be a disadvantage in terms of pharmacists’ time because they are very busy in chemists, aren't they? Very busy pharmacists’. (P17, female, >70 age group)

Because of these challenges, some of the participants proposed an alternative model whereby medicines would be dropped off in a specified area within a pharmacy without the need to speak to any staff. Those medicines would be despatched to a clinical centre where a pharmacist or trained technician completes a quality check in an ‘off‐site’ model. An additional idea was to repackage returned medicines before returning them to pharmacies for reuse. However, the costs associated with having an off‐site system were highlighted as potentially prohibitive. Some participants thought that pharmaceutical companies should be obliged to support medicines reuse processes financially or even help in the repackaging process. For example:

Medicines have labels on them, so one assumes that if you gave them back to the pharmacy, for example, he would then have to send them back to the supplier, the supplier would have to send them back to the manufacturer, the manufacturer would then have to repackage them, and then they have to come all the way back down the chain. (P12, female, 60–69 age group)

Incentives were thought to encourage patients to return unused medicines instead of unsafe disposal practices. Incentives could include a points reward system to encourage medicines return or a discount to be offered on any medicines reused.

So pharmacist can probably say here we are Mr. X, here is those returned tablet and they are 50 pence instead of £1 or whatever it is. So that sort of thing. (P14, male, 60–69 age group)

Accordingly, a working definition of medicines reuse as a behaviour coalesced as:

accepting prescribed medication with more than 6 months of shelf‐life remaining that, as verified by a pharmacist, had been kept untampered for less than three months, under normal storage conditions and in an original sealed blister pack, by another patient before being returned to a community pharmacy.

People taking part in medicines reuse behaviour were seen as:

adult patients prescribed medication for a chronic (not terminal) condition with the capacity to consent.

## Discussion

A working definition of medicines reuse as a behaviour was produced. In addition, people's ideas about the advantages and disadvantages of medicines reuse, who might approve or disapprove of medicines reuse, and factors that would impede or facilitate medicines reuse were mapped systematically using thematic analysis. The principle findings were the potential for medicines reuse to impact positively on the deleterious economic and environmental impact of medicines waste, juxtaposed against a range of stability and safety risks identified with reusing returned medicines. While participants had trust in pharmacists’ competence to quality‐assure returned medicines, they expressed concerns about their availability and access to sufficient storage space to support medicines reuse processes. Environmentalists and the Green Party were seen as the main proponents of medicines reuse behaviour with drug manufacturers and beneficiaries, some taxpayers and those caring for children seen as the main opponents – there were contradictory views about the stance of the elderly. The physical characteristics of reused medicines, and quality assurance and logistics of medicines reuse processes were considered as factors that enabled or obstructed engagement in medicines reuse.

One of the strengths of the current study is the application of thematic analysis to summarise key themes and to formalise views that the general public hold about medicines reuse, which had only been reported anecdotally and to the authors’ knowledge not appropriately investigated until now. Themes obtained in this study have defined what people understand by medicines reuse behaviour as well as behavioural, normative and control beliefs. These are the domains that according to the TPB are relevant for predicting whether people intend to reuse medicines.^15^ This psychologically driven approach is another strength of the current study which provides a mechanism for measuring people's intentions to engage in medicines reuse behaviour, with a further potential for this approach to be useful in wanting to change people's intentions in the future. However, the views are not likely to be representative of the general UK population, firstly because thematic analysis was completed with a small sample of 19 participants and secondly, because the sample was a self‐selected subgroup of the local population who responded to a call to discuss medicine reuse.

There has been little work carried out previously at examining perceptions about medicines reuse in the UK, apart from a study that examined whether pharmacists from one Health Board in South East Wales could come to some consensus on the barriers and potential solutions towards medicines reuse.^14^ The results showed that pharmacists would be willing to redistribute medicines if certain criteria were met such as being solid dosage forms with a tamper evident seal. Our findings are in line with this. Other criteria expressed by pharmacists^14^ included liability protection, guidance from the professional regulator, that reused medicines must be supplied in new packaging, that technologies would need to be developed to indicate inappropriate storage and that there must be public engagement on medicine redistribution. Our work was completed independently of the above study^14^ and addresses the feasibility of medicines reuse from the perspective of the general public, without whose approval medicines reuse could not become a reality.

The participants interviewed recognised the problem of medicines waste and the potential for medicines reuse to minimise waste in the future. However, in identifying particular groups that might disapprove of medicines reuse, this study highlights the need to take account of vulnerable patient groups, and to address political challenges if medicines reuse were to become a reality. For example, the stance of the Association of the British Pharmaceutical Industry who represent the pharmaceutical industry in the UK remains unexplored. In addition, the participants expressed positive views about the involvement of pharmacists in the medicines reuse process, which needs to be explored by pharmacy funding, professional and regulatory bodies. Interestingly, the people in this study commented only on financial incentives for patients and not for pharmacists. An alternative model not requiring community pharmacies to quality check medicines for reuse was also suggested in this study, which partly mimics the American medication collection system.^13^ However, US legislation dictates for ‘A state‐licensed pharmacist or pharmacy to be part of the verification and distribution process’.^13^ The logistic of medicines reuse in the UK therefore needs to be further explored. Concerns about tampering and counterfeit medicines entering the medicines reuse supply chain might be addressed when the European Union directive on falsified medicinal products (2011/62/EU) comes into force in the UK in 2019, since a supplementary Delegated Regulation (EU2016/161) requires marketing authorisation holders to add tamper evidence and a unique identifier to the outer packaging of medicinal products.^19^ The role of heat, light and moisture sensitive monitoring labels as a means of addressing concerns about the degradation of returned medicines during storage remains to be investigated. Resolving the logistics of medicines reuse in the UK could also support the international work of charities such as InterCare^20^ that rely on donated medicines.

The next steps are to develop and test a formal questionnaire that can capture systematically nationwide views of medicines reuse and people's willingness to reuse medicines in the future.

## Conclusion

This study suggests that people could potentially agree to reuse medicines that are returned to pharmacies if their concerns about safety and quality of the returned medicines are addressed, the physical characteristics of medicines are satisfactory, and the medicines reuse process is well defined and managed. This is a qualitative study with a small number of participants recruited from one local area in the UK meaning that the results are not necessarily generalisable. The themes generated will enable a structured questionnaire to be developed for quantifying broader nationwide views about medicines reuse and people's intention to reuse medicines in the future.

## Declarations

### Conflict of interest

The Author(s) declare(s) that they have no conflicts of interest to disclose.

### Funding

This research is part of a PhD project. The PhD student, HA, is sponsored and funded by Al‐Zarqa University (under the regulation of the Jordanian Ministry of Higher Education).

### Authors’ contributions

PD (the primary supervisor) and HA contributed to the conception and design of the study, data collection, analysis, interpretation of data, revisions to the manuscript and final approval of the version to be published. NP made contributions to the supervision of HA and revisions to the manuscript and final approval of the version to be published. All Authors state that they had complete access to the study data that support the publication.

## Supporting information


**Table S1.** Standards for reporting qualitative research (SRQR).

**Appendix S1.** The recruitment advert placed in the university’s community newsletter in spring 2016.
