# Supplementary material for: How do people conceptualise the reuse of medicines? An interview study
Source: Int J Pharm Pract. 2017 Aug 9;26(3):232–41. doi: 10.1111/ijpp.12391 (PMC5969265; doi:10.1111/ijpp.12391)

Could medicines be reused?

Do you currently use, or have you in the past used, medicines on a regular basis for an ongoing illness (for example, asthma, blood pressure, diabetes)? If so, are you willing to speak to a researcher, at the University of Reading's School of Pharmacy, about your views on the reuse of medicines?

Medicine reuse is the idea that medicines returned by one patient can be dispensed by a pharmacist to another patient (instead of disposal as waste – which is what currently takes place).

Your views are important to this study. If you would be willing to be interviewed on this subject at our Whiteknights campus, we can reimburse you for reasonable travel expenses and offer you a £10 Amazon voucher for your time.

Please contact:
Hamza Alhamad
h.q.m.alhamad@pgr.reading.ac.uk

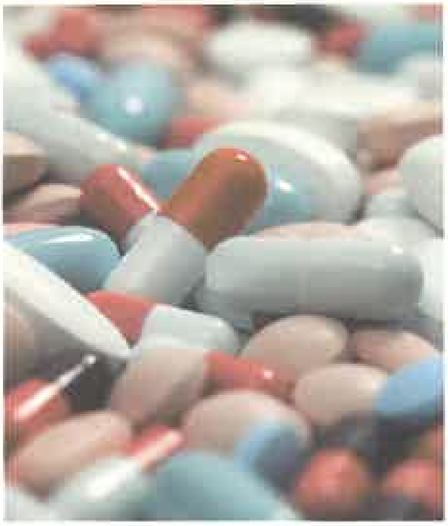

Is cocoa good for brain function?

At the University of Reading we are looking for healthy non-smokers aged 60–75 years who would like to take part in a study investigating the long-term effects of a cocoa-based supplement on cognitive performance and the brain.

The study is taking place over 36 weeks during which you would be required to consume a cocoa-based supplement or placebo daily for 24 weeks. However, you will only need to attend the University on three mornings to provide a blood sample, complete some questionnaires and computer-based tasks, and undergo ultrasound of an artery in your upper arm as well as brain imaging. You'll need to arrive fasted but food will be provided during the visit. In addition, you will be asked to bring a 24-hour urine sample with you.

We would be very grateful for your assistance and you will be compensated for your time and travel expenses

Eligibility to participate would be determined following a screening visit, but if you are generally healthy and do not take medication for blood pressure or diabetes, we would love to hear from you.

Please contact Anja or Georgina from the research team:
CoCo@reading.ac.uk
mobile: 07580 237963

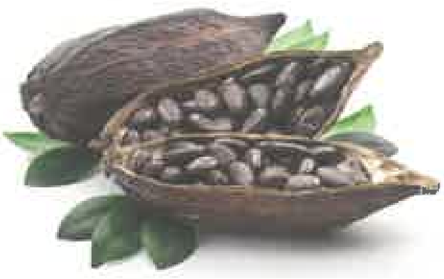

Supplement: Supplementary file 2 — Appendix S1. The recruitment advert placed in the university’s community newsletter in spring 2016. [file IJPP-26-232-s002.pdf]
